# The effects of time of day and chronotype on cognitive and physical performance in healthy volunteers

**DOI:** 10.1186/s40798-018-0162-z

**Published:** 2018-10-24

**Authors:** Elise R. Facer-Childs, Sophie Boiling, George M. Balanos

**Affiliations:** 1 School of Sport, Exercise and Rehabilitation Sciences, University of Birmingham, Edgbaston, Birmingham, B15 2TT UK; 2 School of Psychology, Centre for Human Brain Health, University of Birmingham, Edgbaston, Birmingham, B15 2TT UK

**Keywords:** Diurnal variation, Chronotype, Performance, Athletes, Sports, Circadian rhythms, Sleep

## Abstract

**Background:**

Whether you are a morning lark or a night owl has proven to be a key contributor in the timing of peak athletic performance. Recent evidence suggests that accounting for these differences, known as one’s chronotype, results in significantly different diurnal performance profiles. However, there is limited research investigating multiple measures of performance simultaneously over the course of a socially constrained day.

**Objectives:**

This study aimed to investigate the impact of chronotype on indices of cognitive and physical performance at different times of day in healthy volunteers.

**Methods:**

We recruited 56 healthy individuals categorised as early (ECT, *n* = 25) or late (LCT, *n* = 31) chronotypes using the Munich ChronoType Questionnaire, circadian phase markers and objective actigraphy. Measures of cognitive and physical performance, along with self-reported daytime sleepiness, were taken at multiple times of day (14:00 h, 20:00 h and 08:00 h the following morning).

**Results:**

Here, we find significantly different diurnal variation profiles between ECTs and LCTs, for daytime sleepiness, psychomotor vigilance, executive function and isometric grip strength. LCTs were significantly impaired in all measures in the morning compared to ECTs.

**Conclusion:**

Our results provide evidence to support the notion that ‘night owls’ are compromised earlier in the day. We offer new insight into how differences in habitual sleep patterns and circadian rhythms impact cognitive and physical measures of performance. These findings may have implications for the sports world, e.g. athletes, coaches and teams, who are constantly looking for ways to minimise performance deficits and maximise performance gains.

## Key Points

- Peak performance differs significantly between early and late chronotypes i.e. ‘larks’ and ‘owls’ in simple and complex measures of cognitive and physical performance. Early chronotypes perform at their best earlier in the day compared to late chronotypes. Late chronotypes have significantly higher daytime sleepiness compared to early chronotypes and perform worse in the morning across all cognitive and physical measures.
- Time since entrained awakening can be used effectively to predict performance profiles in healthy volunteers during the day across measures of cognitive and physical tasks.
- Our findings could be of significant interest for elite performance settings, e.g. military, first responders (firefighters, police officers, paramedics) and in particular, the sports world, by providing coaches, managers and teams a greater understanding of how to achieve an advantageous edge over competitors.

## Background

The fundamental objective of performance research is to maximise physical and mental performance through identifying methods that create marginal gains. At the highest level of competition, athletic success is decided on the thinnest of margins and the demand for determining new ways in which to obtain an advantage remains highly desirable. A potential biological source that can influence performance is the time of day [1] and individual differences in circadian timing [2]. These endogenously driven near 24 h circadian rhythms are controlled by the suprachiasmatic nucleus (SCN), which is situated in the anterior hypothalamus [3]. Neural and hormonal outputs from the SCN drive a multitude of behavioural and physiological rhythms, with notable factors being temperature regulation [4], hormonal release [5, 6] and gene expression [7]. Circadian rhythms are also synchronised/entrained by exogenous factors such as light and social signals [8–10].

An individual’s predisposition towards the morning or evening, commonly termed one’s chronotype, can be grouped into either early chronotypes (ECT), late chronotypes (LCT) or those in between (intermediate chronotypes) [11]. ECTs, or ‘larks’, have significantly early sleep-wake cycles compared to LCTs, or ‘night owls’, who prefer to function later in the day. These differences are not only observed in sleep patterns but also in multiple physiological [12], behavioural [11] and genetic [13] oscillations that occur over a near 24 h period.

The effect of circadian rhythms on sporting performance is well documented, and there is evidence to suggest that the rhythmicity of physiological and behavioural processes is correlated with peak performance times. The majority of current research suggests that optimal athletic performance occurs in the late afternoon-early evening, coinciding with the peak of core body temperature (CBT acrophase; 16:00–18:00) [14]. In contrast, performance is suggested to be impaired when CBT is at its lowest (CBT nadir; 03:00) [4]. A higher core body temperature has been shown to facilitate actin-myosin cross bridging in skeletal muscle and thus is thought to be associated with enhanced physical performance [15]. A number of studies have shown that muscular strength, independent of the muscle group or contraction speed, peaks in the late afternoon/early evening. Similar peaks have also been reported for anaerobic exercise and short-term power (see [16] for a review).

Although much of the research into the physical elements of athletic performance have been shown to occur during the early evening, there has been controversy surrounding the diurnal variation of cognitive performance in sports. Accuracy has been reported to be better during the morning [17], along with measures such as fine motor control and short-term memory [18]. However, other technical skills investigated in tennis, swimming and soccer have been shown to be better in the afternoon or evening [16].

Overall, very few studies have been able to measure multiple elements of cognitive and physical performance simultaneously, bringing to light the need to study combinations of measures [19]. On top of this, time of day is rarely taken into account, suggesting the need to investigate how multiple measures of performance are impacted by time of day.

To add to the lack of consideration of diurnal variations, the majority of research on the effect of time of day on cognitive and physical performance in sport fails to investigate, or control for, the potential impact of chronotype. More recently, diurnal performance profiles have been studied between ECTs and LCTs in order to expose whether there is significant variation when individual differences in circadian timing are taken into account. Rowing performance measured in the morning and the evening revealed significantly better performance in the morning for ECTs, although this difference was only ~ 1% [20]. These findings have been supported when evaluating diurnal variation in aerobic performance between ECTs and LCTs [21]. Peak aerobic performance for ECTs was found to be significantly earlier in the day (12:00 h), ~ 5/6 h after entrained waking, compared to that of LCTs whose peak occurred considerably later (20:00 h), ~ 11 h post entrained waking. Furthermore, while ECTs showed a 7.6% variation in performance across the day, the variation shown by LCTs was substantially greater at 26.2%. This study opened new insights into how individual differences, such as circadian phenotype, can impact on athletic performance and suggests that LCTs are more sensitive to diurnal fluctuations in performance. The prevalence of LCTs in elite sports is estimated to be ~ 10% [22], a value much lower than the estimated ~ 40% in the general population [23]. This observation could reflect that a lower number of LCTs are reaching national/international level, suggesting chronotype as a potential factor that determines the path to a successful athletic career.

It is well known that athletic performance is defined by the characteristics of a complex network of mental and physical elements. Whilst there is evidence for the effect of chronotype on aerobic performance, investigations on the effect of time of day on a range of performance elements for different chronotypes is still lacking. This presents an interesting and timely opportunity to study multiple measures of performance simultaneously to allow a more holistic view of the impact of chronotype on performance. Therefore, the present study was designed with the aim of providing a more rounded approach to the effect of chronotype on performance in healthy volunteers by combining cognitive and physical tasks across multiple experimental sessions reflecting a ‘normal real world day’ (08:00–20:00 h). We hypothesise that chronotype, as well as time since entrained awakening, will be indicative of the time of day at which peak performance occurs.

## Methods

### Participants

The study was approved by the University of Birmingham Research Ethics Committee and was performed in accordance with the 1964 Declaration of Helsinki. Assessment for chronotype was performed in 261 healthy individuals using corrected mid-sleep on free days (MSF_sc_) from the Munich ChronoType Questionnaire (MCTQ) [11]. Participants were selected based on no prior diagnoses of sleep, neurological or psychiatric disorders, were not taking any medications that affect sleep and did not have any physical impairment that would prevent them completing a simple handgrip task. Individuals who were categorised as ‘early’ (MSF_sc_ less than 04:00 h) or ‘late’ (MSF_sc_ greater than 05:00 h) were invited to take part in the main study. A total of 56 healthy individuals (33 female, 21.8 ± 3.8 years) categorised as ECTs (*n* = 25, age 22.8 ± 4.5 years, 16 female, MSF_sc_ = 02:38 h ± 00:07) or LCTs (*n* = 31, age 20.8 ± 3.0 years, 17 female, MSF_sc_ = 06:59 h ± 00:12) took part. Participants gave written informed consent before involvement, all details provided were given on a voluntary basis and participants were free to withdraw at any time. A subset of 38 participants (16 ECTs and 22 LCTs) provided saliva samples for melatonin and cortisol rhythm analysis and underwent actigraphy throughout the study to validate chronotype groups (Table 1). Participants followed their normal preferred routines and were not confined to particular schedules for a 2-week baseline period, following which they attended the laboratory for testing sessions at 14:00 h, 20:00 h and 08:00 h the following morning.

**Table 1:** Summary of demographic data, sleep and physiological variables for early (ECT) and late (LCT) chronotypes

Variable measured (mean ± SEM)	ECTs	LCTs	Significance
Sample size	*N* = 25	*N* = 31	n/a
Number of testing sessions	*N* = 75	*N* = 93	n/a
Number of males/females	M = 9	M = 14	*p* = 0.59 (ns)^b^
	F = 16	F = 17	
Age (years) (mean ± SD)	22.8 ± 4.5	20.8 ± 3.0	*p* = 0.084 (ns)^a^
Height (cm)	172.5 ± 1.7	172.5 ± 1.9	*p* = 0.99 (ns)^a^
Weight (kg)	67.7 ± 2.3	69.8 ± 2.0	*p* = 0.55 (ns)^a^
MSF_sc_ (hh:mm)	02:38 ± 00:07	06:59 ± 00:12	*p* < 0.0001^a^
Sleep onset (hh:mm)	23:03 ± 00:07	02:36 ± 00:14	*p* < 0.0001^a^
Wake-up time (hh:mm)	06:45 ± 00:08	10:30 ± 00:14	*p* < 0.0001^a^
Sleep duration (h)	7.69 ± 0.14	7.85 ± 0.14	*p* = 0.55 (ns)^a^
Dim light melatonin onset (hh:mm)	20:27 ± 00:16	23:55 ± 00:26	*p* < 0.0001^a^
Cortisol peak time (hh:mm)	07:04 ± 00:16	11:13 ± 00:23	*p* < 0.0001^a^

Values are shown as mean ± SEM unless specified. The significance is shown with ^a^unpaired two-sample *t* tests, ^b^Fisher’s exact test. MSF_sc_ = corrected mid-sleep time of free days. All *p* values are corrected for the false discovery rate

#### Power Calculations

The sample size requirements were calculated using G*Power [24], with an alpha of 0.05 and a power of 0.90 for two-tailed independent (two groups) *t* tests. Based on previously published performance results between ECTs and LCTs [21] assuming a between-group standard deviation of 6.83%, a total sample size of 52 participants were necessary to detect significant changes (effect size = 0.83).

### Sleep Analysis

Wrist actigraphy can be used to provide a reliable and accurate overview of sleep/wake patterns and behaviour over long periods of time. Actiwatches, which have been validated against some PSG parameters such as total sleep time, sleep efficiency and wake after sleep onset [25], were developed to monitor 24 h activity of individuals in their home environment and provide a cheaper and easier alternative to methods like PSG [26]. These small wrist-worn devices are triaxial accelerometers and contain 4D motion sensors which can detect and record both light and activity/movement in given time frames, e.g. epochs of 2 s up to 1 min, to distinguish between activity and lack of, which is assumed to be, sleep. Actiwatches are used extensively in sleep and circadian research as well as having more clinical uses in respiratory medicine, mental health and other fields [27].

Actigraphs (Actiwatch® Light, AWLs, 2006, Cambridge Neurotechnology Ltd) were worn on the non-dominant wrist for 13–16 days to monitor sleep and activity patterns in the participants’ natural environment. Data were acquired in 1-min epochs, confirmed with daily sleep diaries and analysed using Sleep Analysis 7 Software (version 7.23, Cambridge Neurotechnology Ltd).

### Physiological Data

Participants provided saliva samples during one morning and one evening during the week of the testing sessions. Radioimmunoassays of melatonin and cortisol in human saliva were performed (Stockgrand Ltd., University of Surrey) using an iodine-125 (I^125^) radioactive labelled tracer and solid phase separation as described previously [28]. Assays were run with quality controls before and after samples.

Individual dim light melatonin onset (DLMO) values were calculated using the mean of the baseline concentration values plus two standard deviations of the mean. This concentration was used to calculate the timing of melatonin onset through a linear response function. Due to insufficient or contaminated samples, DLMO values were unable to be calculated for two ECTs and four LCTs. Cortisol peak was calculated as the time of the highest cortisol concentration recorded.

### Performance Measures

#### Cognitive

Cognitive testing consisted of a two minute psychomotor vigilance task (PVT) [29] and a ~ 10 minute executive function (EF) task called the Memory and Attention Test (MAT, V2.1, Team Focus Limited, https://teamfocus.co.uk/wp-content/uploads/2016/11/MAT-Users-Guide-2013.pdf).

The PVT has been widely used in multiple fields of research as well as clinically [29, 30]. It is a simple reaction time paradigm which uses visual stimuli at random intervals and is the most widely used cognitive performance test in sleep and circadian research [31]. It requires the subject to look at a blank screen and respond whenever a stimulus is presented. Numerous studies have linked both long (10 min) and short versions (2 min) of the PVT to sleep deficits, e.g. sleep deprivation [32, 33] as well as circadian disruption [34, 35]. The PVT is therefore a useful tool to capture an element of waking performance in studies involving the time of day and circadian/sleep patterns. The MAT contains measures of speed, accuracy, memory and decidedness, which combined gives a measure of cognitive executive function. Reliability indices are 0.89 (internal consistency) and 0.77 (test re-test). The version used in this study was a shortened version designed to be sensitive to short-term time of day changes and easily repeatable. In the EF task, participants were presented with a screen containing a number of different coloured shapes and asked to complete a task based on a rule (e.g. click on all the blue diamonds). Following this, a subsequent more complex rule was given (e.g. click on all the red circles unless there is a black square). There were a total of five different rules with five trials for each that needed to be completed for a total of 25 trials of increasing complexity requiring a higher level of cognitive processing due to the need to retain information required for the task. Reaction time values from the PVT and time to completion in the EF task were taken as an index of cognitive performance.

All participants completed the cognitive tests three times during the baseline period in their home environment and compliance was monitored remotely. Results from the trials show no significant difference between trials two and three suggesting a plateau had been achieved, thereby minimising learning effects (*p* > 0.05).

#### Physical

Maximum voluntary contraction (MVC) was measured using the 6 second isometric grip strength test using an electronic hand dynamometer (EH101, CAMRY) [36]. Participants were standing with their elbow fully extended and used their dominant hand in a pronated position to apply as much grip pressure as possible on the handgrip dynamometer for 6 seconds. The maximum value was recorded in kilograms. This process was repeated three times with 2 minute intervals in between. To ensure consistent motivational feedback to participants, a strict protocol was adhered to with a script being read out each time to encourage the participant to try their best.

#### Sleepiness

Self-reported daytime sleepiness was measured using the Karolinska Sleepiness Scale (KSS). The KSS gives an indication of current sleepiness and is one of the most widely used scales for measuring self-reported sleepiness. It consists of a simple 9-point Likert scale, from 1: very alert to 9: fighting sleep, in which the participant is asked to indicate their feelings of sleepiness during the 5 min prior to completing the rating. It takes less than a minute to complete. The KSS has been validated against objective measures of sleepiness (alpha and theta EEG activity) [37] as well as the Karolinska Drowsiness tests, alpha attenuation tests and performance variables such as the PVT [38].

### Statistical Analysis

Statistical comparisons of behavioural data between the ECT and LCT groups were performed using GraphPad Prism (version 7.00 for Windows, GraphPad Software, La Jolla, CA, USA). Non-parametric tests were implemented where data did not follow a normal distribution. To control for multiple comparisons, all *p* values were corrected for false discovery rate using the Benjamini-Hochberg methods [39]. Diurnal variations were analysed using two-way analysis of variance (ANOVA) for repeated measures with post hoc multiple comparison tests, adding chronotype (early/late) and time of day (08:00 h, 14:00 h and 20:00 h) as factors. After exploring different nonlinear curve fits, diurnal variations in performance and sleepiness variables were plotted using second-degree regression curves. Time since awakening values were calculated as number of hours from habitual wake-up time for each participant. Significance levels are displayed as ns = not significant, *p* < 0.05 = *, *p* < 0.01 = **, *p* < 0.001 = ***, *p* < 0.0001 = ****. Values are represented as the mean ± standard error of the mean (SEM) unless specified otherwise. Exact *p* values are given to two significant figures, apart from when significance is identified as less than 0.0001, in which case *p* < 0.0001 is reported.

## Results

Table 1 shows the demographic, sleep and circadian characteristics of participants included in each group (ECTs and LCTs). There were no significant differences in age, height, weight or sleep duration. The groups differed significantly in MSF_sc_, sleep onset, wake-up time, DLMO and cortisol peak time (all *p* < 0.0001).

### Karolinska Sleepiness Scale (KSS)

**Fig. 1:**
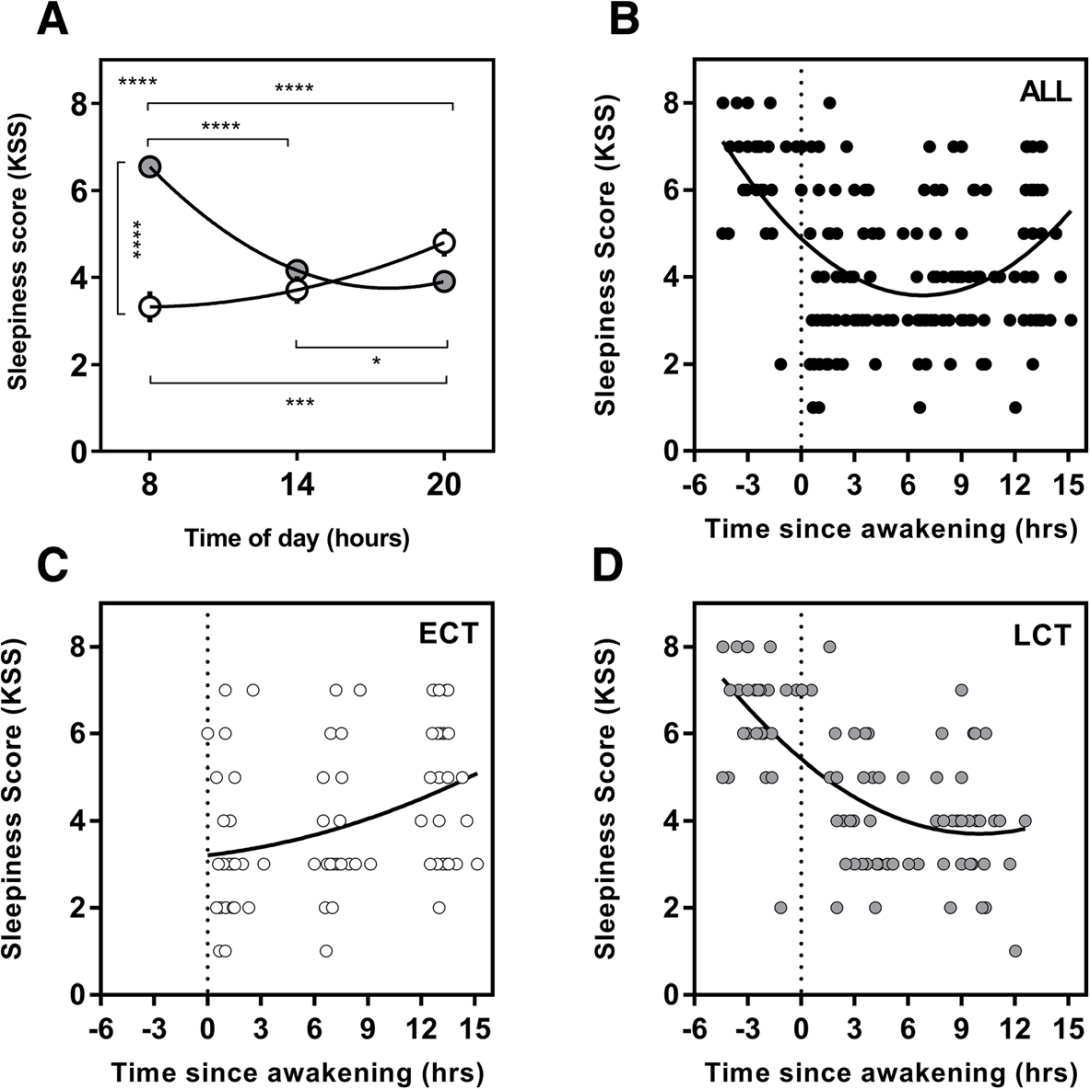
Diurnal variation in daytime sleepiness (KSS) and sleepiness as a function of time since awakening. Clock time variation (**a**) in KSS between ECTs (white circles) and LCTs (grey circles). KSS as a function of time since awakening for all subjects (*n* = 56; **b**), ECTs (*n* = 25; **c**), LCTs (*n* = 31; **d**). Entrained wake-up time, i.e. biological time 0, is shown with the dotted line. Curves are second-order polynomial non-linear regressions. The significance is shown as * = *p* < 0.05, ** = *p* < 0.01, *** = *p* < 0.001, ****p < 0.0001. The result shown in the upper left of (**a**) plots represents the interaction between the time of day and chronotype derived from the overall two-way ANOVA. Post hoc test results are shown with lines and asterisk between the time of day and chronotype groups

There was a significant interaction of time of day and chronotype for daytime sleepiness, as measured using the KSS (*F*(2, 106) = 31.7, *p* < 0.0001). A significant main effect was found for time of day (*F*(2, 106) = 5.9, *p* < 0.004) and chronotype (*F*(1, 53) = 17, *p* = 0.0001) (Fig. 1). Significant diurnal variations were found for ECTs between morning and evening (*p* = 0.0007) as well as afternoon to evening (*p* = 0.04). Sleepiness in LCTs also showed significant diurnal variations from morning to afternoon and evening (both *p* < 0.0001). Post hoc tests between the groups revealed that sleepiness at 08:00 h was significantly higher for LCTs compared to ECTs (*p* < 0.0001).

### Psychomotor Vigilance Task (PVT)

**Fig. 2:**
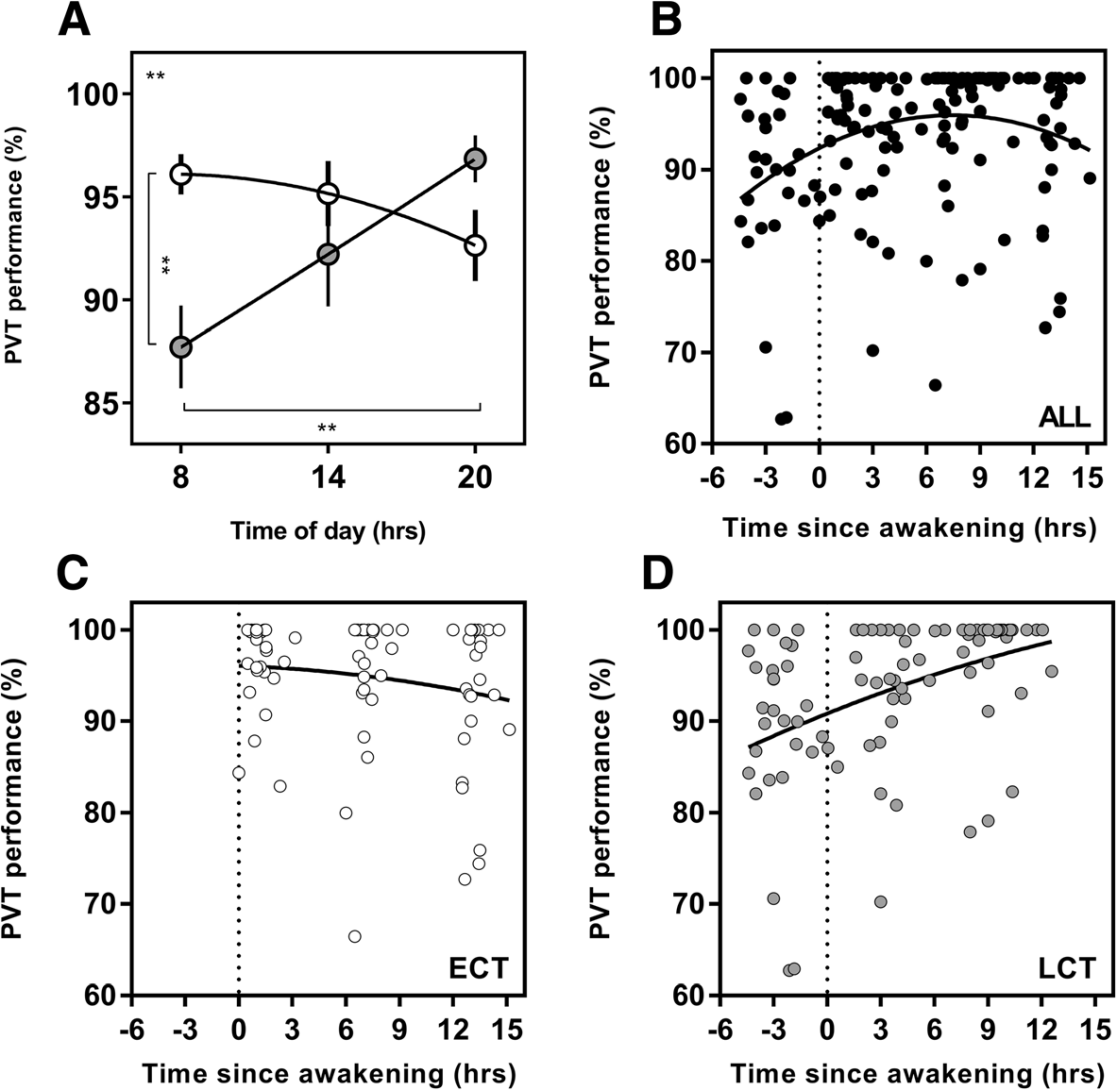
Diurnal variation in psychomotor vigilance (PVT) and PVT as a function of time since awakening. Clock time variation (**a**) in PVT performance between ECTs (white circles) and LCTs (grey circles). PVT as a function of time since awakening for all subjects (*n* = 56; **b**), ECTs (*n* = 25; **c**), LCTs (*n* = 31; **d**). Data are expressed as the percentage of individual personal best performance. Entrained wake-up time, i.e. biological time 0, is shown with the dotted line. Curves are second-order polynomial non-linear regressions. The significance is shown as * = *p* < 0.05, ** = *p* < 0.01, *** = *p* < 0.001, ****p < 0.0001. The result shown in the upper left of (**a**) plots represents the interaction between the time of day and chronotype derived from the overall two-way ANOVA. Post hoc test results are shown with lines and asterisk between the time of day and chronotype groups

A significant interaction between time of day and chronotype was found for PVT performance (*F*(2, 106) = 5.7, *p* = 0.004) (Fig. 2). There was a significant diurnal variation in PVT performance for LCTs with morning being significantly worse than evening (*p* = 0.001) but not for ECTs. Diurnal changes in performance were 3.5% for ECTs and 9.1% for LCTs. At 08:00, ECTs performed 8.4% better than LCTs (*p* = 0.004).

### Executive Function (EF)

**Fig. 3:**
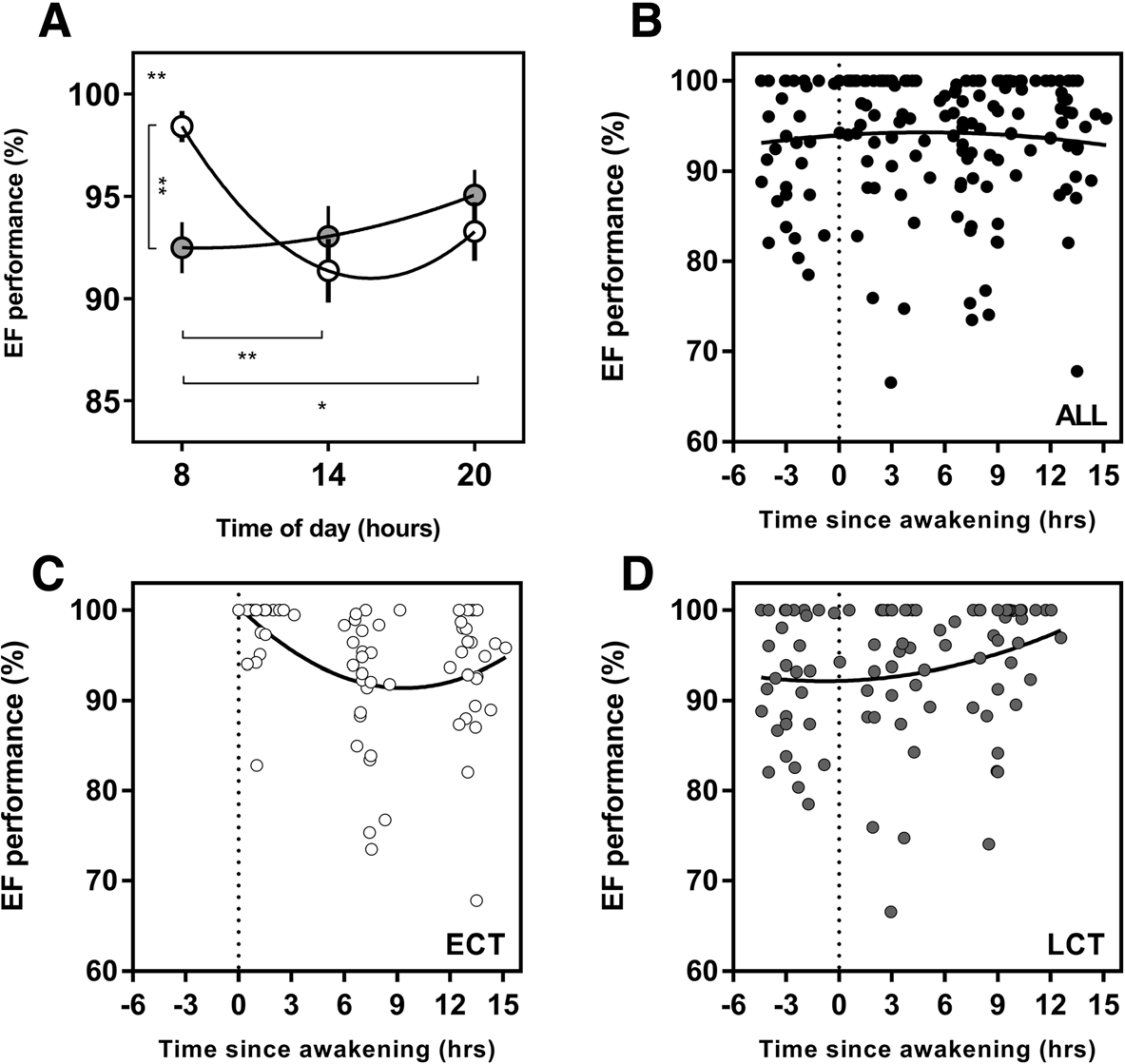
Diurnal variation in executive function (EF) and EF as a function of time since awakening. Clock time variation (**a**) in PVT performance between ECTs (white circles) and LCTs (grey circles). EF as a function of time since awakening for all subjects (*n* = 56; **b**), ECTs (*n* = 25; **c**), LCTs (*n* = 31; **d**). Data are expressed as the percentage of individual personal best performance. Entrained wake-up time, i.e. biological time 0, is shown with the dotted line. Curves are second-order polynomial non-linear regressions. The significance is shown as * = *p* < 0.05, ** = *p* < 0.01, *** = *p* < 0.001, ****p < 0.0001. The result shown in the upper left of (**a**) plots represents the interaction between the time of day and chronotype derived from the overall two-way ANOVA. Post hoc test results are shown with lines and asterisk between the time of day and chronotype groups

A significant interaction between time of day and chronotype was found for EF performance (*F*(2, 108) = 5.5, *p* = 0.005) (Fig. 3). There was a significant diurnal variation in PVT performance for ECTs with morning performance being significantly better than the afternoon (*p* = 0.002) and evening (*p* = 0.03). Diurnal changes in performance were 7.1% for ECTs and 2.6% for LCTs. At 08:00, ECTs performed 5.9% better than LCTs (*p* = 0.006).

### Maximal Voluntary Contraction (MVC)

**Fig. 4:**
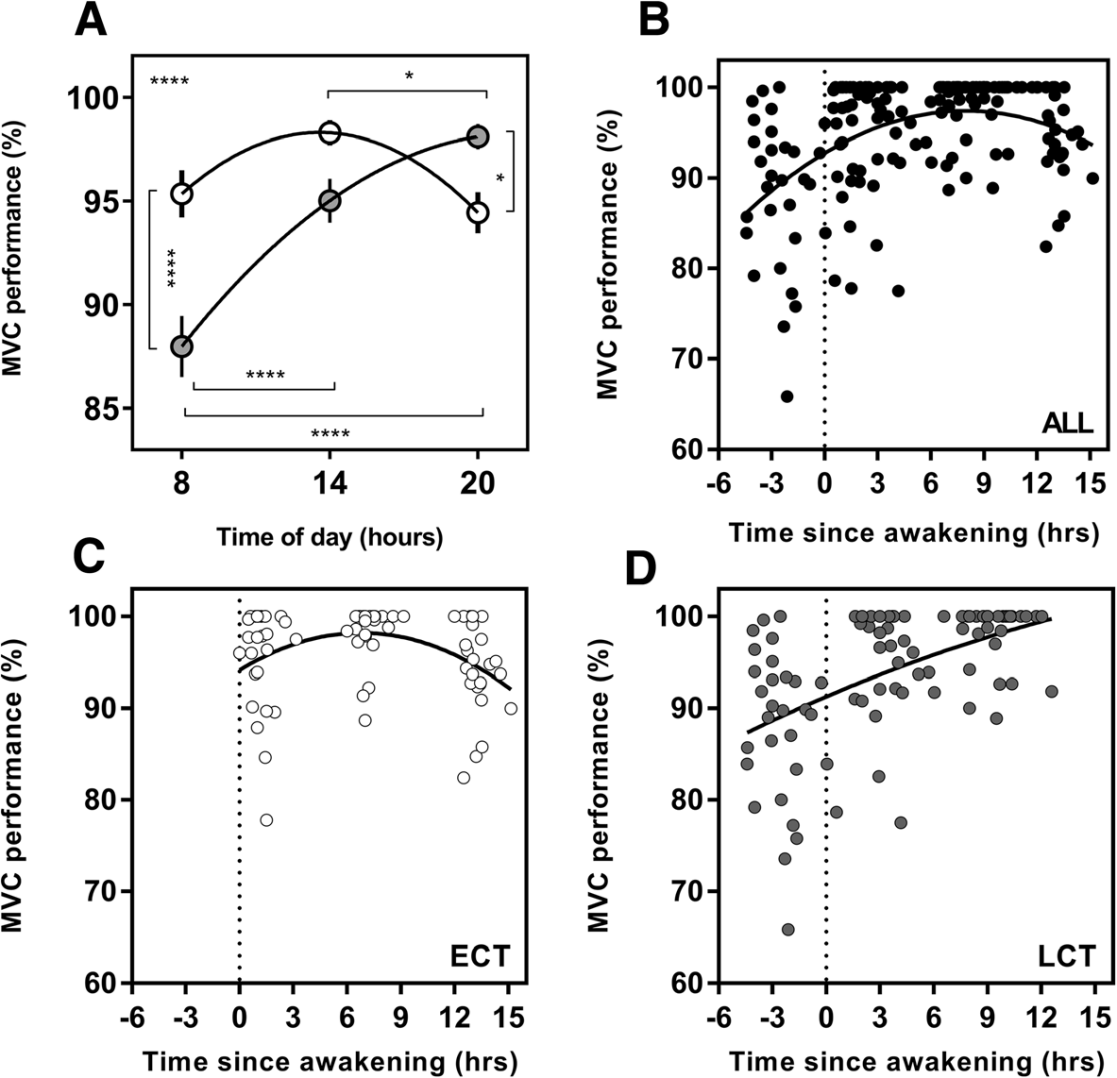
Diurnal variation in maximum voluntary contraction (MVC) and MVC as a function of time since awakening. Clock time variation (**a**) in PVT performance between ECTs (white circles) and LCTs (grey circles). MVC as a function of time since awakening for all subjects (*n* = 56; **b**), ECTs (*n* = 25; **c**), LCTs (*n* = 31; **d**). Data are expressed as the percentage of individual personal best performance. Entrained wake-up time, i.e. biological time 0, is shown with the dotted line. Curves are second-order polynomial non-linear regressions. The significance is shown as * = *p* < 0.05, ** = *p* < 0.01, *** = *p* < 0.001, ****p < 0.0001. The result shown in the upper left of (**a**) plots represents the interaction between the time of day and chronotype derived from the overall two-way ANOVA. Post hoc test results are shown with lines and asterisk between the time of day and chronotype groups

A significant main effect of time of day (*F*(2,108) = 13.8, *p* < 0.0001), chronotype (*F*(1,54) = 7.28, *p* = 0.009) and the interaction between time of day and chronotype (*F*(2, 108) = 13.8, *p* < 0.0001) was found for MVC performance (Fig. 4). There was a significant diurnal variation in MVC performance for both ECTs (3.8%) and LCTs (10.1%). ECTs were significantly better in the afternoon compared to the morning (*p* = 0.04). LCTs were significantly worse in the morning compared to both the afternoon and evening (*p* < 0.0001). At 08:00, ECTs performed 7.4% better than LCTs (*p* < 0.0001), whereas at 20:00, LCTs performed 3.7% better than ECTs (*p* = 0.04).

### Performance as a Function of Time Since Entrained Awakening

When analysing the data as a function of time since awakening (i.e. number of hours since entrained wake-up time), different peak performance times were revealed between the groups. Within the constraints of the model, whole group PVT performance occurred 7.4 h after wake-up time. However, the peak ECTs was at biological time 0 (at the time of entrained wake-up time) and 12.6 h after wake-up time for LCTs (Fig. 2). The same was seen for EF performance, with whole group peak occurring 4.9 h after entrained wake-up time, but for ECTs, this occurred at 0.2 h, and for LCTs, it occurred at 12.6 h after wake-up time (Fig. 3). Peak MVC performance was 8.0 h after entrained wake-up time for the whole group. Within each chronotype group, peak performance was 6.7 h after entrained wake-up time for ECTs and 12.6 h after entrained wake-up time for LCTs (Fig. 4).

## Discussion

Competitive athletes are placed under substantial pressure to perform at their best due to considerable advancement in sports science support, technology and rewards for medal positions from competitions [40]. It is therefore crucial that any potential enhancement in performance which may provide a competitive edge is thoroughly explored. Multiple studies have uncovered a link between chronotype and physical performance enhancement [20, 21, 41, 42], supporting the idea that time of day and individual differences in circadian timing may impact diurnal variation across other measures of performance [43]. However, to our knowledge, this is the first study to use multiple indices of cognitive and physical performance in regard to chronotype and time of day.

Using a multifactorial approach to explore the different factors and variables which may affect enhancement in performance, our results showed significant differences between ECTs and LCTs in sleepiness, as well as across multiple cognitive performance measures, e.g. reaction time (PVT)and executive function (EF). Furthermore, we show that a simple physical performance measure (maximal voluntary contraction of isometric grip strength), exhibits a significant diurnal variation between the two groups. This is consistent with the results on a more complex measure of physical performance, namely, cardiovascular endurance [21]. Importantly, our results uncovered that ECTs performed significantly better than LCTs across all performance measures in the morning (08:00 h). The morning testing session took place when LCTs would usually be sleeping, which supports previous research claims that completing tasks during one’s ‘biological night’ can be detrimental to performance [44]. This finding has since solidified the importance of chronotype identification within athletes.

Chronic misalignment is generally associated with differences between an individual’s endogenous circadian system and external time cues [45]. Typically, LCTs experience chronic misalignment due to following an earlier schedule during the ‘work week’ and reverting back to later sleeping patterns on ‘free days’. Commonly known outcomes of this circadian misalignment, such as jet lag or night shift work, are known to negatively impact on health and performance [46].

### Time of Day, Chronotype and Daytime Sleepiness

Our results showed LCT’s subjective sleepiness at 08:00 h (Fig. 1) was significantly higher than ECTs at the same time, with a three-point difference on the KSS to distinguish between ‘alert’ and ‘some signs of sleepiness’. This could be an important consideration for LCTs, who often have to ‘perform’ before their entrained/habitual wake-up time. This is particularly important in professions such as pilots, medical professionals, military personnel, commercial drivers and other occupations whereby a reduction in alertness and decision-making capabilities may be consequentially life-threatening [47]. This desynchronization is also prevalent within an athletic environment, whereby an athlete may be required to travel across time zones to compete. If the misalignment is not corrected prior to competition, an athlete’s decision making, alertness and executive functions may be hindered, resulting in non-optimal performance [48].

### Time of Day, Chronotype and Measures of Performance

Optimised cognitive abilities are essential to the basic functioning and have also been recognised as an important component of successful athletic performance [49, 50]. PVT reflects the attentional state of an individual [51], and research has shown that a better PVT score is associated with improved response time and accuracy in interceptive sports, such as tennis and squash, as well as improved response accuracy in strategic sports such as field hockey and soccer [52]. Executive function incorporates cognitive factors including working memory, problem-solving and decision-making, taking place in the prefrontal cortex [53]. A significant correlation has been found between the level of sporting ability and success rate in completing executive function tasks [54]. Further research has also shown that self-paced athletes, such as swimmers and runners, perform better at inhibition tasks. In contrast, externally paced sportspeople, such as rugby and soccer players, score higher on problem-solving tasks [55]. As a result of these findings, it is clear that the multiple aspects of cognition are imperative to an athlete in order to achieve maximum and well-rounded performance.

One of the key findings from this study highlights that cognitive performance is significantly impaired in LCTs when they are required to perform both simple and complex tasks during the morning. We have shown that LCTs are compromised at 08:00 h in both the PVT and EF tasks, with performance being significantly worse than ECTs by 8.4% and 5.9%, respectively. Coupled with the significantly higher ratings of sleepiness at 08:00 h, this is consistent with research that partial sleep deprivation can result in an increased response time and higher number of lapses when undertaking the PVT [33, 56]. Interestingly, there were significant diurnal variations in PVT performance for LCTs but not for ECTs. However, when looking at a more complex measure of cognitive performance during the EF task, this relationship was reversed. Although a non-significant but gradual improvement was seen across the three testing sessions in LCTs, it was only ECTs that showed significant diurnal variations (Fig. 3). A potential reason for this could be attributed to the complexity and nature of the task. Two recent meta-analyses have shown that sleep deprivation has greater negative impact on the performance in simple cognitive tasks, such as the PVT, compared to more complex cognitive tasks [57, 58]. Both papers attribute this to the higher degree of boredom and lower arousal that is associated with simple tasks and suggested that these factors may be amplified due to a lack of sleep. More complex tasks, however, require and generate greater engagement and stimulation, therefore potentially accounting for the detrimental effects of sleep loss on performance. Our results show that LCTs have a damped amplitude in diurnal variation for the EF task, suggesting a potential compensatory reaction during a more complex task. This finding aligns with the research that suggests simple tasks are more adversely affected by sleep deprivation [57]. The fact that the amplitude of diurnal variations seems to be impacted by the nature of tasks, i.e. simple vs complex, as well as between chronotypes, presents an interesting area for future research.

It is well established that elements of physical performance, particularly those involving muscular strength, tend to peak in the early evening [18]; however, much research has failed to consider the impact of chronotype. Our study showed that ECTs performed their best MVC at 14:00 h, whereas the peak for LCTs was at 20:00 h (Fig. 4).

### Performance as a Function of Time Since Entrained Awakening

Time since entrained wake-up has been proposed as a predictor of peak performance in aerobic endurance tasks, with the peak for LCTs occurring significantly later compared to ECTs [21]. This finding is consistent with the measures of performance shown in our study in healthy volunteers. The main observations relating to chronotype and performance since the time of awakening are (1) peak performance in ECTs always occurs closer to the habitual wake-up time compared to LCTs. (2) For ECTs, measures of cognitive performance are best almost immediately after entrained wake-up time, whereas measures of physical performance peak between ~ 5/6 h and ~ 7 h after wake-up time for aerobic [21] and MVC, respectively. (3) Regardless of the measure of performance, LCTs do not reach their peak until at least 12 h after entrained wake-up time. This suggests that LCTs have a much narrower window of opportunity to perform at their best during the course of a typical day, which could have significant implications for athletes with a late chronotype who are required to train and compete earlier than their biological peak.

Our findings support much of the current literature by suggesting clear differences in performance profiles between ECTs and LCTs. These results complement those previously recorded with more complex measures of physical performance [20, 21, 42] by showing similar trends of significantly better performance from ECTs in the morning, as well as very different peak times as a function of time since entrained awakening.

### Implications for Performance

Knowledge of the potential impact that an individual’s chronotype may have on both cognitive and physical performance could have significant beneficial implications within the general population. This information could also provide new insight to sectors that require individuals to achieve optimal performance such as military personnel, first responders (firefighters, police and paramedics) and professional athletes. At an elite sporting level, a winning margin can be as little as 1%. At the most recent 2016 Olympic Games (Rio de Janeiro, Brazil), had the fourth place swimmer in the men’s 100 m freestyle improved his time by 0.5% (0.24 s), it would have been enough to secure a gold medal. Similarly, if the last placed competitor in the men’s 100 m sprint had run 0.25 s faster (2.5%), it would have been enough to beat Usain Bolt. These differences are so minute that any potential advantage to be gained should be researched in depth. Previous research has shown that aerobic performance can vary up to 26% over the course of a day [21]. The present study supports this by showing a ~ 10% diurnal increase in a simple measure of physical performance and ~ 9% and ~ 7% variation in simple and complex measures of cognitive performance, respectively. Using these findings, training strategies could be developed and implemented by coaches to maximise performance through adhering to the athlete’s individual chronotype and taking into account the time since entrained awakening. This would be of particular relevance to LCTs who have been shown to exhibit greater variation in diurnal performance profiles. If LCTs are required to train/compete during non-optimal morning hours, it could significantly influence their abilities since our study shows that LCTs are compromised at 08:00 h. These results do not just apply for individuals but also teams, as previous research has shown that chronotype distribution within a team is highly predictive of overall performance, highlighting how this research can be used to give teams a ‘circadian advantage’ [41]. However, rigid schedules often present a challenge for athletes having to perform at non-optimal times. Therefore, this information could be used to develop personalised interventions targeting at sleep and circadian biology aimed to shift the timing of peak performance to accommodate inflexible competition times. It is fair to speculate from our results that such a strategy may result in greater improvements in overall performance, although in-depth research is required to develop these approaches.

### Limitations

The purpose of testing at clock times as opposed to internal biological time was to investigate how these two groups behave during the hours of a ‘normal day’ (08:00 h to 20:00 h). This data can therefore be translated into real-world settings and hold implications for monitoring performance. The disadvantages of this design are that it cannot separate the number of influences affecting the outcomes or separate the effects of the circadian system from the sleep homeostat. To explore truly circadian effects, strict laboratory-based protocols, e.g. constant routine or forced desynchrony, over 24 h or more would be required. However, it could be argued that the combined approach is more applicable to the real world as behaviour and performance are ultimately impacted by both factors, and the more controlled protocols could result in poorer external validity due to the unrealistic settings.

Here, we investigated a relatively simple measure of physical performance using isometric grip strength, and thus, we restrict the ability to determine how more complex measures would be affected [31]. The performance itself is multifaceted and cannot be defined by one mechanism alone. Internally, physical performance can be measured by physiological markers such as hormones levels (e.g. cortisol, melatonin, and testosterone), CBT, heart rate, respiratory rate and maximum oxygen uptake. External physical performance can be described and monitored through strength, power, aerobic capacity, anaerobic capacity and specialist skills tailored to certain sports such as accuracy. These varied and complex processes cause the research of ‘performance’ to be viewed differently by clinicians, psychologists, sports scientists and others.

The sample population was predominately either competitive athletes or individuals who engaged with sport on a regular basis (84% of ECTs and 81% of LCTs). Therefore, we believe that the sample studied here is a representative cohort for the athletic implications that we suggest in the manuscript. The choice of not seeking to recruit specifically elite athletes was made on accounting for practical difficulties in doing so, but more importantly in order to allow the best possible compliance and accurate, reliable data collection. The reality of the daily schedule of elite athletes would present many constraints that would limit the ability to carry out this study design accurately. For example, athletes tend to follow very strict training and competition schedules, which do not allow them to sleep/wake according to their biological preference. All participants in this study followed their preferred routines for the duration of the testing period, thereby allowing us to gain a true indication of their chronotype without ‘masking’ effects of imposed schedules. The study design also required participants to provide saliva samples and attend the laboratory for multiple testing sessions, something that would be difficult for elite athletes to comply with. Carrying out real-world research in healthy volunteers is necessary in order to inform populations such as elite athletes; but also, because this study design investigates elements that contribute to athletic performance and not athletic performance per se, the findings of the study are relevant for a wider range of physically active individuals.

Therefore, it must be acknowledged that the measures tested and the sample population used in this study could restrict the ecological validity. However, our results show a strong similarity to previously published work on aerobic capacity in athletes [21], and previous studies have shown measures of muscle strength do correlate with sprint and jump performance [59].

## Conclusion

In summary, our study has highlighted the influence that chronotype has on the diurnal variation of cognitive and physical performance measures. We show that LCTs are significantly impaired during the morning hours in all measures of performance compared to ECTs. Moreover, we show that the time since entrained awakening can be used as a predictor of peak performance. These findings add considerably to the current body of literature by providing a more holistic approach to quantifying performance. These findings should elicit extensive interest in elite performance settings e.g. sports, military and emergency services, as well as in the scientific and wider community, as accounting for chronotype has been shown to have a substantial impact across all the measures used. This should highlight to coaches, managers, athletes and organisations that exploiting an individual’s chronotype and the time of day within training and competition regimes should play a key role in unlocking the full potential of athletes and achieving marginal gains over competitors.

## Abbreviations

DLMO: Dim light melatonin onset
ECT: Early chronotype
EF: Executive function
KSS: Karolinska Sleepiness Scale
PVT: Psychomotor vigilance task
LCT: Late chronotype
MSFsc: Corrected mid-sleep on free days
MVC: Maximum voluntary contraction
SCN: Suprachiasmatic nucleus
